# Identification of a marker for two lineages within the GC1 clone of *Acinetobacter baumannii*

**DOI:** 10.1093/jac/dkt379

**Published:** 2013-09-29

**Authors:** Mohammad Hamidian, Matthew Wynn, Kathryn E. Holt, Derek Pickard, Gordon Dougan, Ruth M. Hall

**Affiliations:** 1 School of Molecular Bioscience, The University of Sydney, NSW 2006, Australia; 2 Department of Biochemistry and Molecular Biology and Bio21 Molecular Science and Biotechnology Institute, The University of Melbourne, Victoria, Australia; 3 Wellcome Sanger Trust Institute, Hinxton, Cambridge, UK

**Keywords:** *A. baumannii*, global clone 1, antibiotic resistance islands, AbaR0, ISAba1

Sir,

Isolates from a collection of 177 antibiotic-resistant *Acinetobacter* recovered at Westmead Hospital, Sydney over the period 1995–2002^1^ were further characterized. The relationships among these isolates had previously been examined by PFGE of ApaI-digested DNA, determination of MICs of several antibiotics and PCR screening to determine whether the *bla*_OXA-23_ gene or class 1 integrons were present.^1^ Eight PFGE pulsotypes were detected. The majority of isolates were *A. baumannii* but five isolates (PFGE pulsotypes III and IV) were not, and these were not examined here. One or two isolates from each year for each remaining pulsotype were tested using triplex PCRs targeting the *oxa-Ab*, *csuE* and *ompA* genes,^2^ to determine whether they belong to global clone 1 (GC1) or global clone 2 (GC2). Pulsotype I isolates were GC1 and isolates from pulsotypes VI, VII and VIII, which first appeared in 1999, were GC2. The 54 GC2 isolates were the only isolates resistant to imipenem and carried the *bla*_OXA-23_ gene.^1^ Types II (*n* = 64) and V (*n* = 2) did not belong to either global clone. This is consistent with a previous report using a smaller number of isolates.^3^

Pulsotype I included 52 isolates and was present in the hospital intensive care unit from 1995 to 1999, with a single isolate recovered in 2000. Isolates in this group are among the earliest multiply antibiotic-resistant *A. baumannii* reported in Australia. They were gentamicin resistant and carried the *aacC1*-orfP-orfP-orfQ-*aadA1* cassette array in a class 1 integron.^1^ They were also resistant to ciprofloxacin but susceptible to third-generation cephalosporins and carbapenems.^1^ Consistent with the GC1 designation, WM98, one of two representatives examined previously, was found here to belong to sequence type ST109 [Oxford multilocus sequence typing (MLST) scheme]. Ten representative isolates, including WM98, covering each of the years 1995–99, were examined in more detail, and, in contrast to the previous report,^1^ WM98 and other members of this group did not include a copy of ISAba1.

The resistance profile was expanded using disc diffusion, and the resistance genes present were determined by PCR.^4^ The *comM* gene was interrupted, and they were found to also carry the *sul1* sulphonamide resistance gene, the *tet*(A) tetracycline resistance determinant and transposon Tn*6020* carrying the *aphA1b* kanamycin and neomycin resistance gene. The *catA1* and *bla*_TEM_ genes were also present. PCR mapping^5^ of WM98 together with sequencing of all IS*26* junctions revealed the presence of a genomic resistance island very closely resembling AbaR3 in AB0057^6^ (GenBank accession number CP001182).

We had noticed that AbaR3 contained a deletion of 108 bp in the 5′-conserved segment (5′-CS) of the class 1 integron, and that this deletion was not present in AbaR5,^4^ which now has been completely sequenced (GenBank accession number FJ172370), or in AbaR1 in AYE (GenBank accession number CU459141) or in AbaR2 in the GC2 isolate ACICU (GenBank accession number CP000863). The location of this deletion is indicated in Figure 1(a). It removes the last 52 bp of the *intI1* gene, and the loss of conserved amino acids in the 16 amino acids replaced at the C-terminus of IntI1^7^ is likely to inactivate it. The deletion appears to have arisen via a rare recombination event or a replication slippage event involving a very short, 8 bp duplication present in the 5′-CS (Figure 1b). Whether the deletion was present in the AbaR of the WM98 group was examined using PCR with primers RH882 (5′-GATGCGTGCACTACGCAAAG-3′) and intI1-RV (5′-GGGCATGGTGGCTGAAGGACC-3′), which generate an amplicon of 1222 bp when the 5′-CS is complete and 1114 bp when the deletion is present and fragments of 659 + 563 or 659 + 455 bp after digestion with BamHI. The 5′-CS is intact in WM98 and related isolates, and this was confirmed by sequencing the PCR amplicon from WM98. This configuration with a complete 5′-CS is likely to pre-date the lineage that includes the deletion, making it the most likely ancestor of AbaR3 and the various AbaR configurations that have arisen subsequently. Therefore, we propose that the AbaR island of WM98 should be named AbaR0. The complete sequence of AbaR0 was assembled^8^ from the whole genome sequence of WM98 determined using Illumina HiSeq, generating 100 bp paired-end reads, and has been deposited in the GenBank DNA database under accession number KF483599.

**Figure 1.**
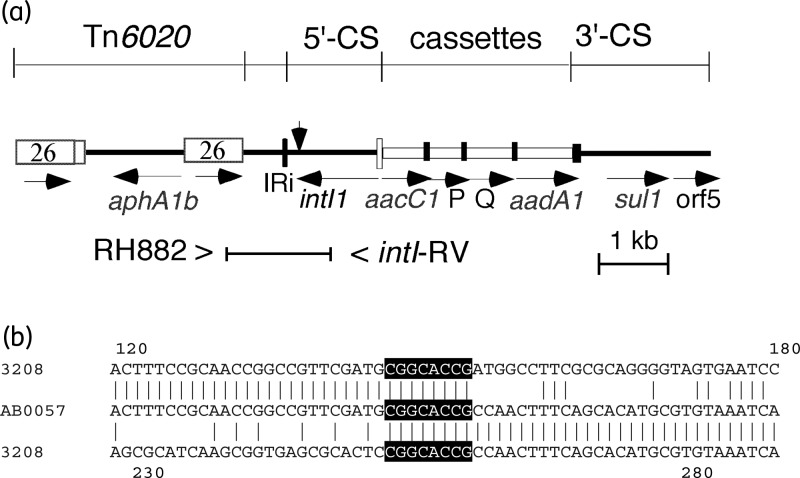
(a) Structure of the part of AbaR islands containing the integron and adjacent Tn*6020*. The positions of PCR primers used to detect the deletion are indicated below, and the vertical arrow indicates the location of the deletion in the 5′-CS. (b) Alignment of the 3208 and AB0057 sequences in the vicinity of the deletion. Identical bases are indicated by vertical lines and bases that occur twice in 3208 are white on black.

As the deletion in *intI1* results from a rare event, it has the potential to assist in tracking different AbaR (i.e. islands in *comM* with a Tn*6019* backbone) and hence GC1 lineages. Therefore its presence in other Australian GC1 isolates was examined. Isolates with an intact 5′-CS and ones with the deletion were both present among these strains. In AbaR21 in the reference GC1 strain RUH875/A297 from 1984,^9^ as well as in AbaR6 and AbaR7 found in isolates from 2006 and 2005,^5^ the 5′-CS is intact. In contrast, AbaR8, found in isolates from 2008,^10^ includes the deletion. Three sporadic isolates from different Australian cities, A85, RBH3 and 6772166, recovered in 2002 or 2003 and described recently as carrying AbaR3,^11^ included the deletion. This deletion, which can be simply detected by PCR, will serve as an additional marker for diverged lineages within the GC1 clone.

## Funding

This study was supported by NHMRC Project Grant APP1026189 and Wellcome Trust grant number 098051. M. H. was supported by a University of Sydney Postgraduate Research Award. K. E. H. was supported by an NHMRC PostDoctoral Fellowship (no. 628930).

## Transparency declarations

None to declare.
